# Supplementary material for: Redefining the role of Ca2+-permeable channels in photoreceptor degeneration using diltiazem
Source: Cell Death Dis. 2022 Jan 10;13(1):47. doi: 10.1038/s41419-021-04482-1 (PMC8748460; doi:10.1038/s41419-021-04482-1)

**Main text Figures**

Das *et al*.: Redefining the role of Ca^2+^-permeable channels in photoreceptor degeneration using diltiazem.

**Figure 1**

**
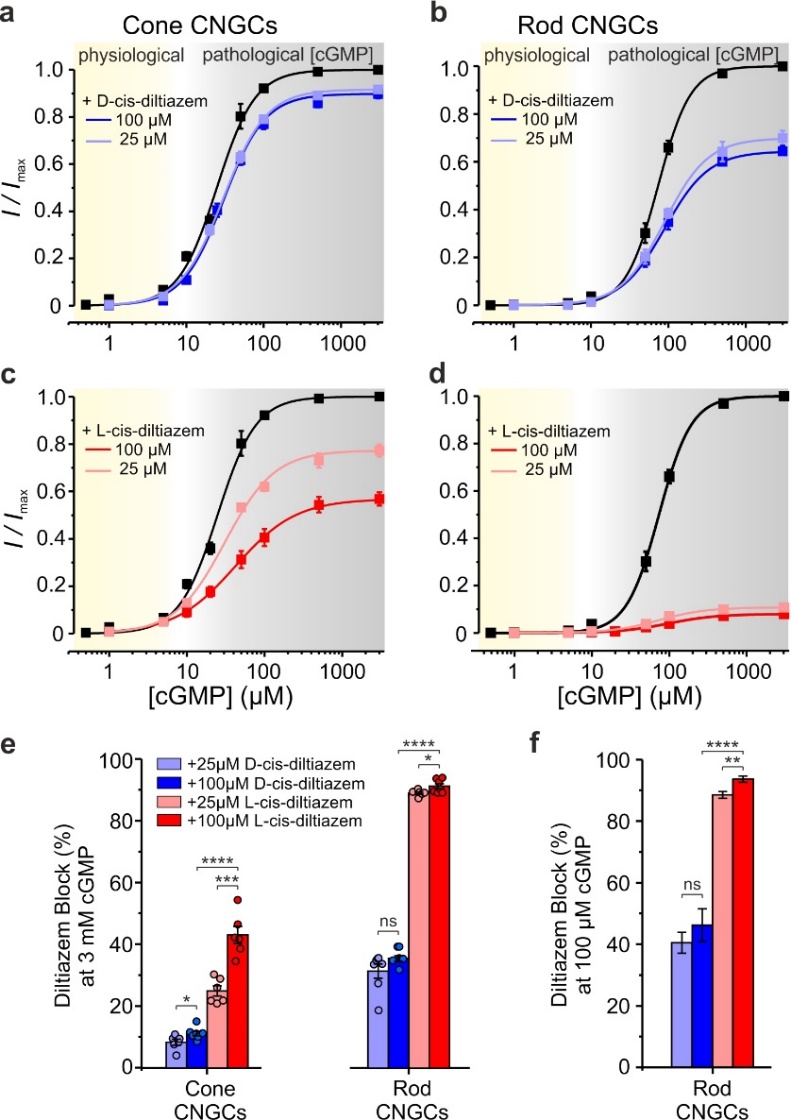
**

**
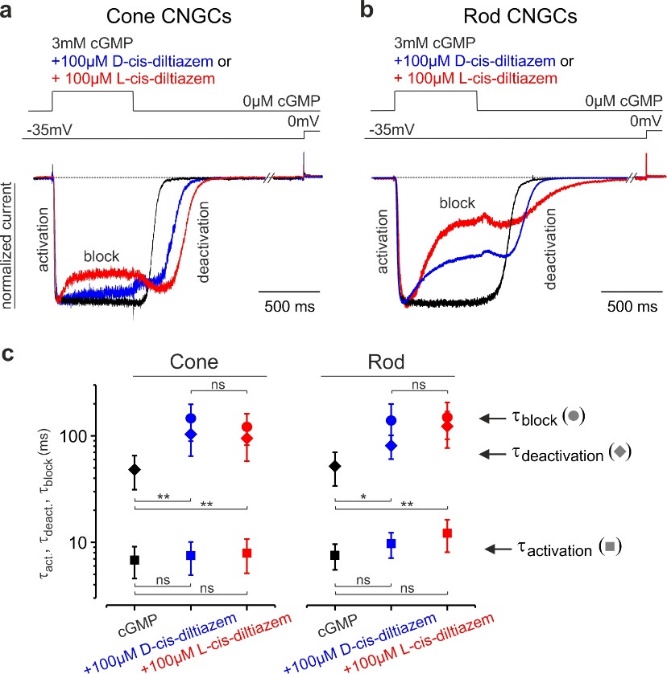
Figure 2**

**Figure 3**

**
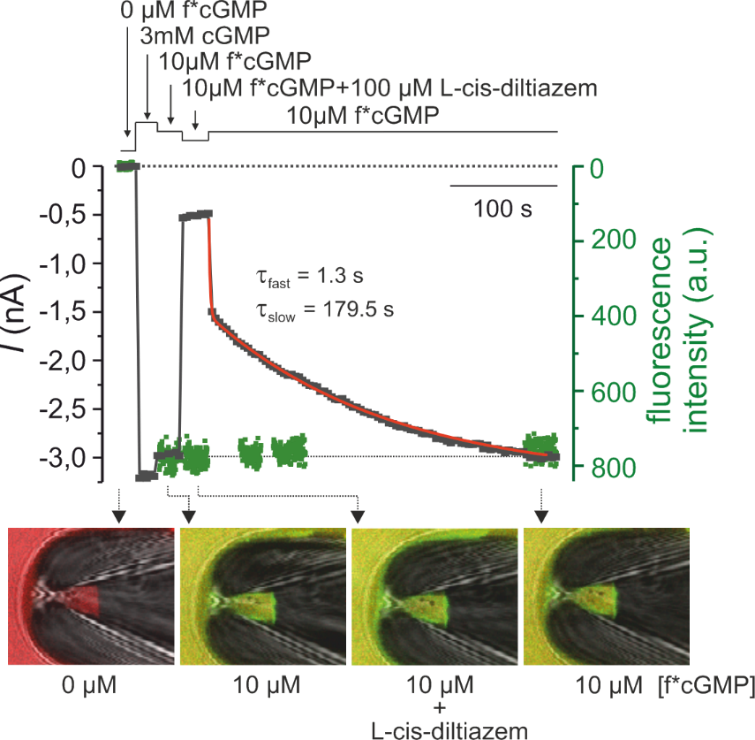
**

**
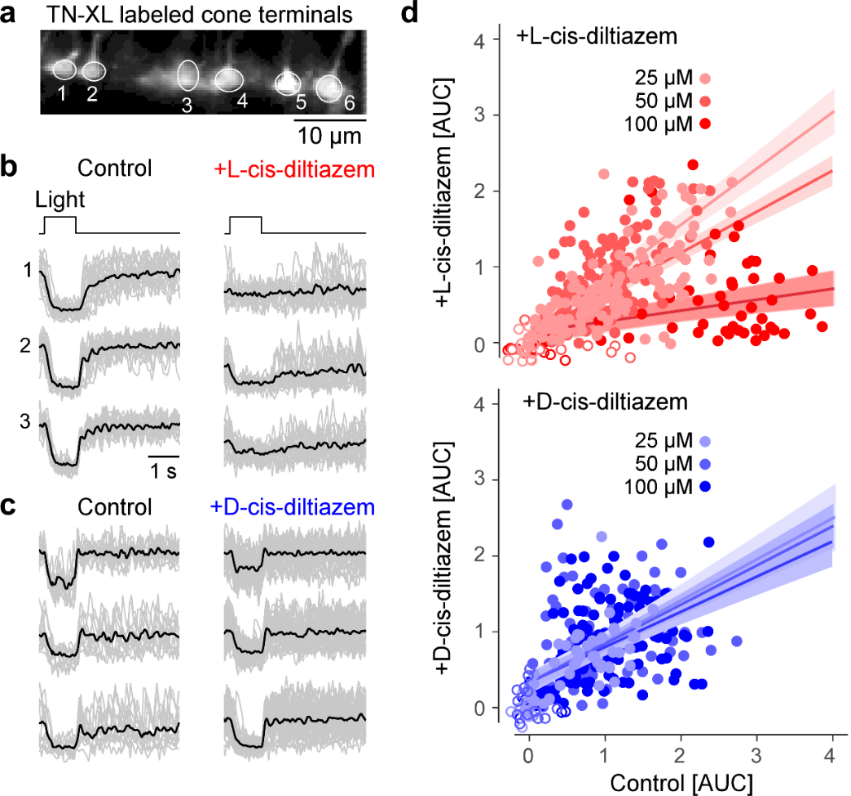
Figure 4**

**Figure 5**

**
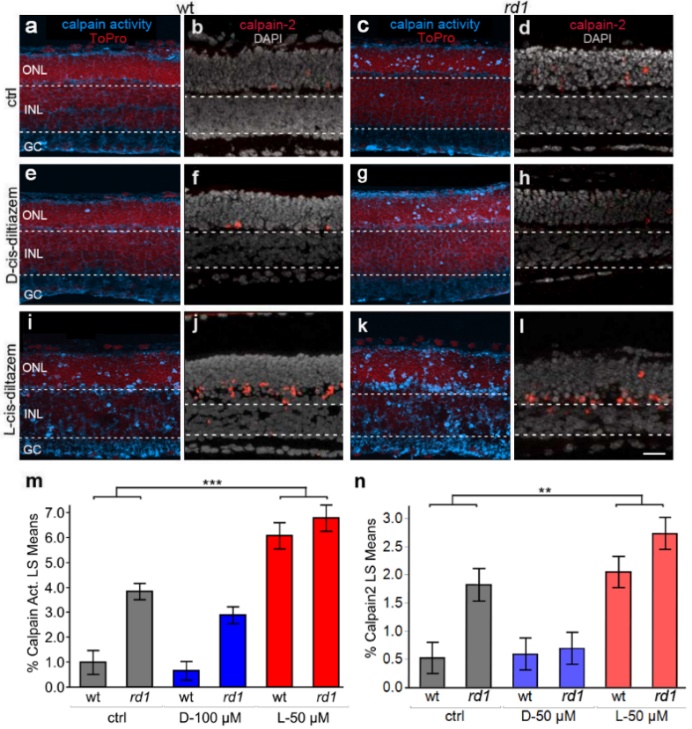
**


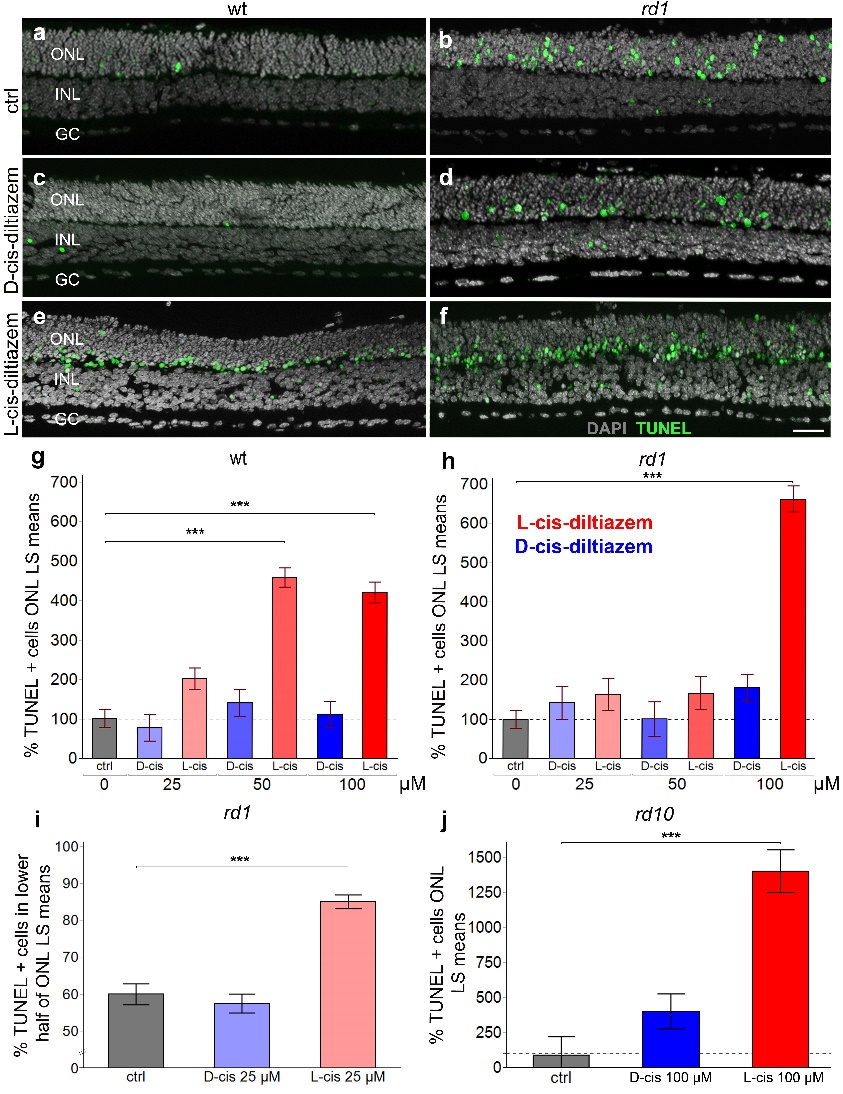
**Figure 6**

**Figure 7**
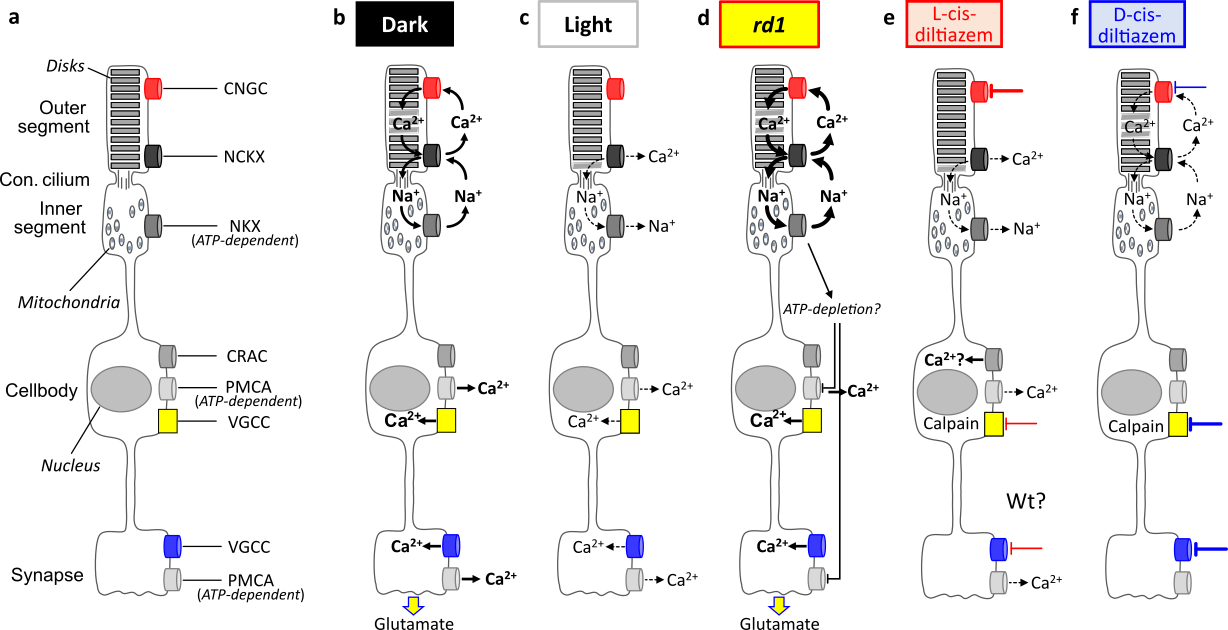

Supplement: Supplementary file 1 — Related Manuscript File [file 41419_2021_4482_MOESM1_ESM.docx]
