# Supplementary material for: Redefining the role of Ca2+-permeable channels in photoreceptor degeneration using diltiazem
Source: Cell Death Dis. 2022 Jan 10;13(1):47. doi: 10.1038/s41419-021-04482-1 (PMC8748460; doi:10.1038/s41419-021-04482-1)
Supplement: Supplementary file 3 — Supplementary material file-collated [file 41419_2021_4482_MOESM3_ESM.pdf]

Supplementary Figures and Tables with Legends

Das *et al*.: Redefining the role of Ca^{2+}-permeable channels in photoreceptor degeneration using diltiazem.

Supplementary Figure S1

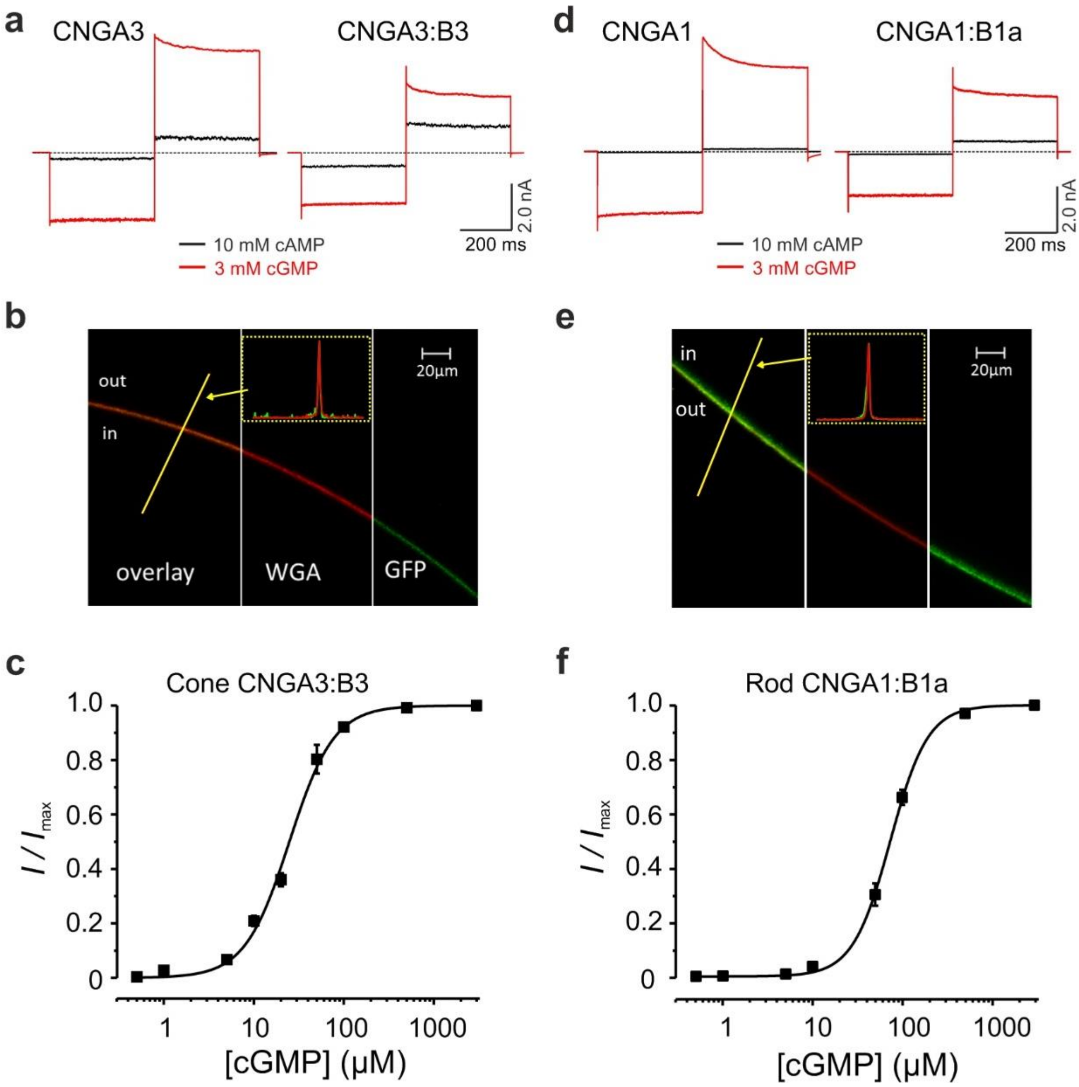

Figure S1: Functional properties of photoreceptor heterotetrameric CNGCs expressed in *Xenopus laevis* oocytes. Representative macroscopic cone (**a**) and rod (**d**) CNGC-current traces from inside-out membrane patches in the presence of 3 mM cGMP (red) and 10 mM cAMP (black). The current traces were elicited by voltage steps from a holding potential of 0 mV to -100, +100 and 0 mV. Leak currents in the absence of cGMP were subtracted for all recordings. For CNGA3 channels the ratio I_{cAMP}/I_{cGMP} was 0.15±0.01 (n=8). CNGB3-subunit incorporation into the CNGA3:B3 channel leads to a significant increase in the cAMP efficacy (I_{cAMP}/I_{cGMP}=0.42±0.03, n=6). Similarly, for CNGA1 channels the ratio I_{cAMP}/I_{cGMP} was 0.019±0.005 (n=12), whereas for heterotetrameric CNGA1:B1a channels the ratio was 0.16±0.02 (n=6). (**b, e**) Representative measurements showing confocal images of oocyte membrane expressing heterotetrameric CNGA3:B3-GFP (**b**) and CNGA1:B1a-GFP (**e**) channels (green fluorescence signal). The oocyte plasma membrane was labelled with Alexa Fluor™ 633 WGA (red fluorescence signal). The small insets show fluorescence profiles along the yellow line, perpendicular to the membrane and confirm the

colocalization of the labelled channels with the oocyte membrane. For each channel isoform we tested more than 10 oocytes from at least two different oocyte batches. (**c**, **f**) cGMP-dependent concentration-activation relationships for cone CNGA3:B3 (**c**) and rod CNGA1:B1a (**f**) channels obtained at -35 mV. The currents triggered by subsaturating ligand concentrations were normalized with respect to the maximal current at 3 mM cGMP. The experimental data points, each representing the mean of 5 to 10 measurements, were fitted with Eq. (1) (see also Table S1).

Supplementary Figure S2

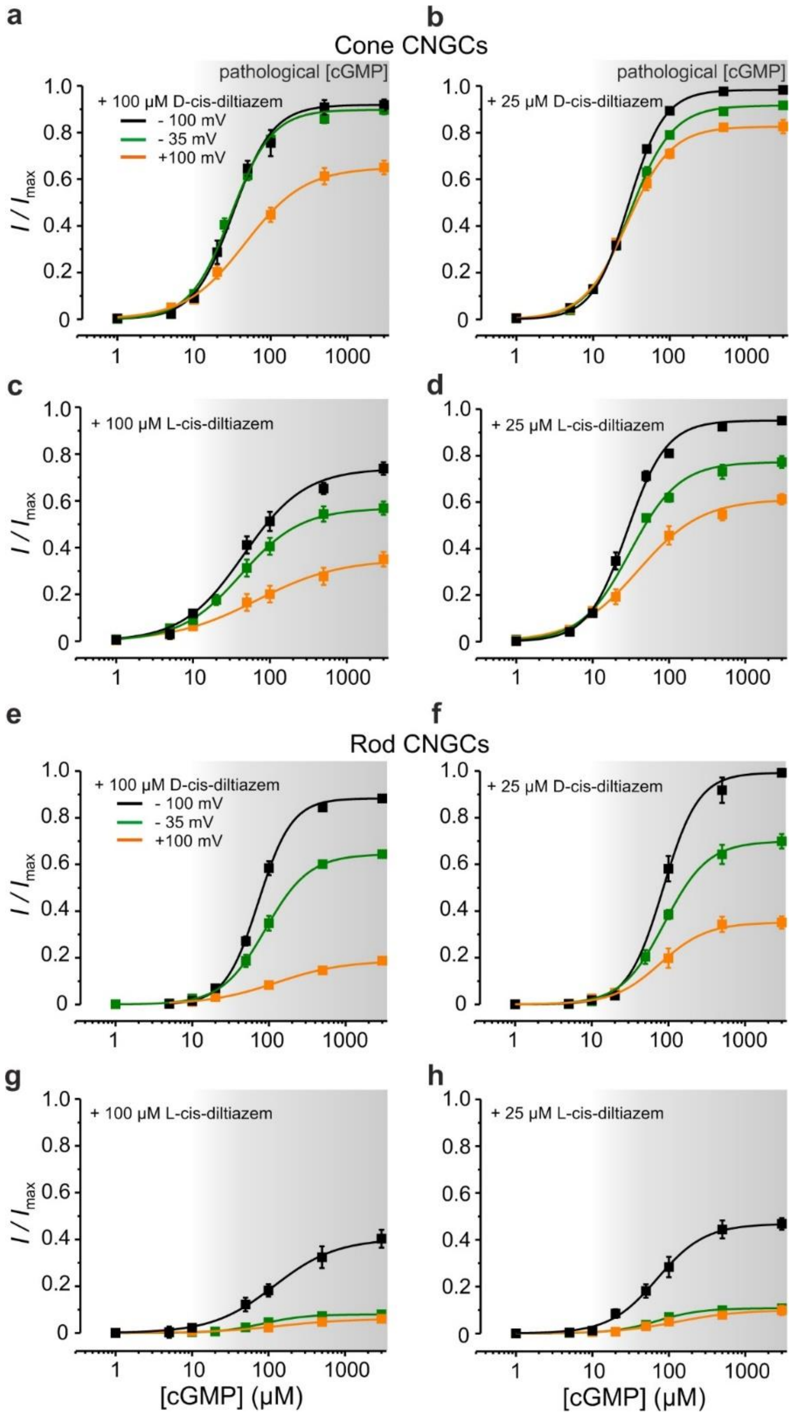

Figure S2: Voltage dependence of D- and L-cis-diltiazem-induced inhibition of photoreceptor CNGCs. cGMP-dependent concentration-activation relationships for cone (**a-d**) and rod (**e-h**) CNGCs in the presence of 100 µM (left) and 25 µM (right) D- and L-cis-diltiazem, respectively, measured at: -100 mV (black symbols), -35 mV (green symbols) and +100 mV (orange symbols). The current amplitudes were normalized with respect to the saturating currents measured in the absence of diltiazem at each individual voltage. The experimental data points were fitted with the Hill equation (Eq. 1). All parameters obtained from the fits are included in Table S1.

Supplementary Figure S3

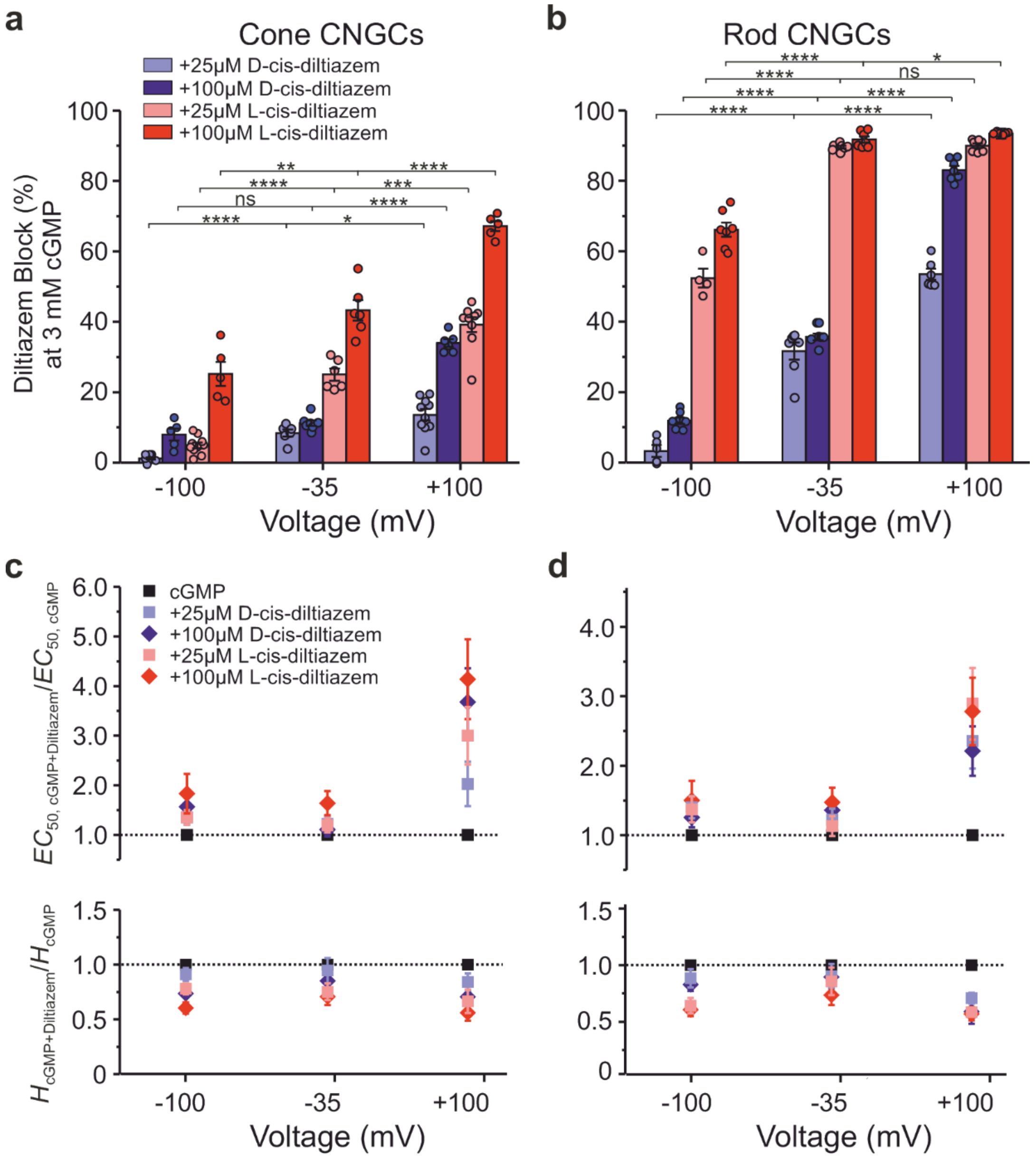

Figure S3: Differential effect of D-cis- and L-cis-diltiazem on CNGC activity and apparent affinity. (**a, b**) D- and L-cis-diltiazem - block of cone and rod CNGC activity triggered by saturating cGMP at three different voltages. The amount of diltiazem block was calculated using Eq. 2. (**c, d**) Effect of D- and L-cis-diltiazem on the channel's apparent affinity. Shown are the $EC_{50,cGMP+Diltiazem}/EC_{50,cGMP}$- and $H_{cGMP+Diltiazem}/H_{cGMP}$-ratios in the presence of 25 µM or 100 µM D- or L-cis-Diltiazem at -100 mV, -35 mV and +100 mV. The EC_{50}- and H-values were obtained from the concentration-activation relationships shown in Figs. 1 and S2 (see also Table S1). For statistical analysis see Table S3.

Supplementary Figure S4

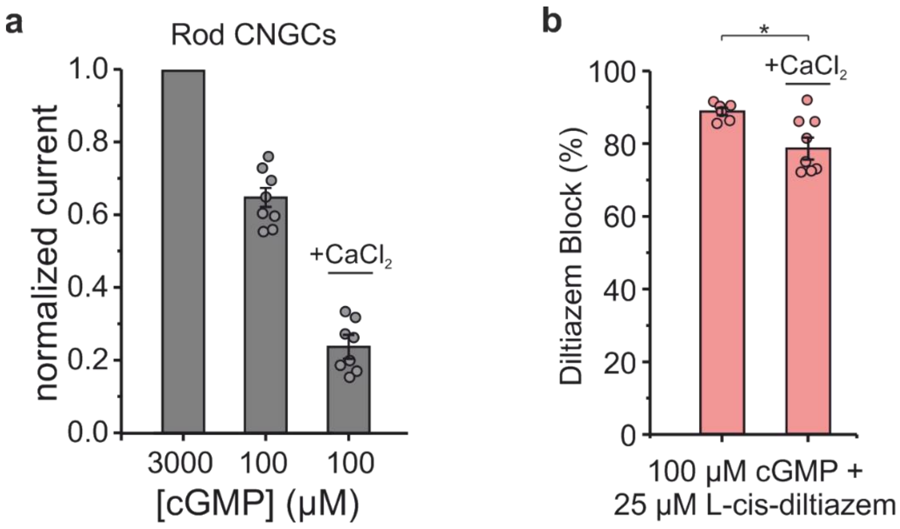

Figure S4: Effect of Ca^{2+} on the blocking effect of L-cis-diltiazem on rod CNGCs. (**a**) The diagram shows normalized rod CNGCs current triggered by 100 µM cGMP, in the absence and in the presence of 1 mM $CaCl_2$ in the extracellular solution. The current at 100 µM was normalized with respect to the current in the presence of 3 mM cGMP, under the respective $CaCl_2$-conditions (n=9). The channel response to cGMP is much weaker in the presence of Ca^{2+} ($I_{cGMP+CaCl2}/I_{max}$ = 0.233±0.03) as it is in its absence (I/I_{max} = 0.65±0.026). (**b**) L-cis-diltiazem - block of rod CNGC activity triggered by 100 µM cGMP in either the presence or absence of Ca^{2+}. The amount of diltiazem block was calculated using Eq. 2. The two-tailed unpaired Student *t*-test was used for the statistical analysis: p = 0.034.

Supplementary Figure S5

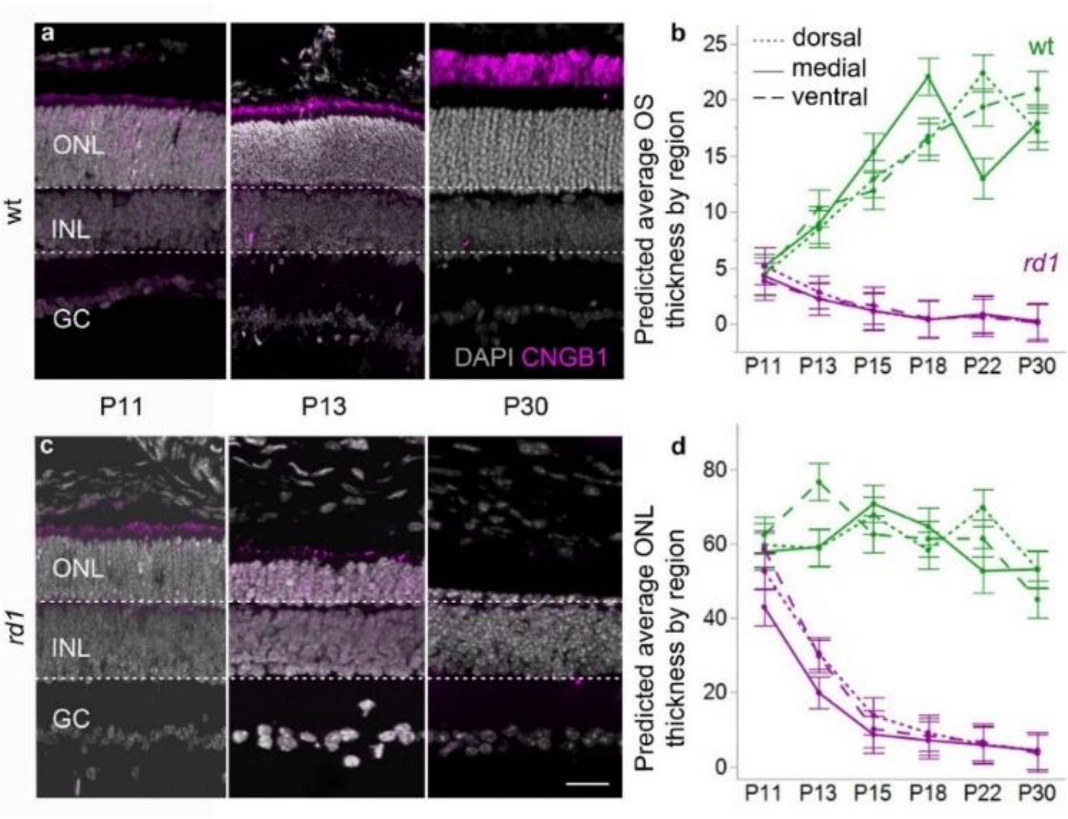

Figure S5: ONL thickness and CNGC expression during *rd1* retinal degeneration. Immunostaining for CNGB1a (magenta) was performed at different post-natal (P) days in wild-type (wt) and *rd1* retina (**a**,**c**). The nuclear counterstain (DAPI, grey) indicates outer nuclear layer (ONL), inner nuclear layer (INL), and ganglion cell layer (GC). Dotted, solid and dashed lines in the graph represent dorsal, medial, and ventral mouse retina respectively (**b**,**d**). (**a**) In wt retina, CNGB1a immunostaining labelled the photoreceptor outer segments, which grew longer from P11 to P30. (**c**) In *rd1* retina, CNGB1a positive outer segments were visible at P11 and P13 but essentially disappeared by P30. (**d**) The thickness of the ONL in wt retina (green) remained approx. constant between P11 and P30, while *rd1* (magenta) ONL size rapidly diminished after P11. (**b**) Outer segments in wt retina grew longer from P11 to P24 until reaching a plateau at a length of approx. 20 µm. In contrast, *rd1* outer segments, while still comparable to wt at P11, had decreased in length to nearly 0 µm by P24. Images and quantification were obtained from retinal sections from 4-5 different animals per time-point and genotype. Scale bar = 30 µm.

Supplementary Figure S6

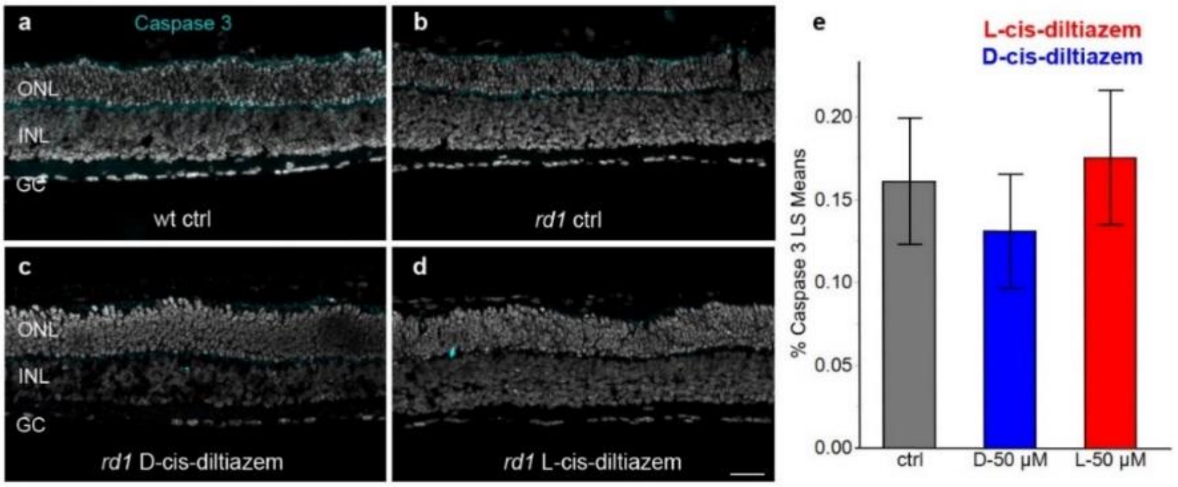

Figure S6: Absence of apoptotic marker during photoreceptor degeneration. Immunostaining for cleaved, activated caspase-3 (turquoise) was performed on *rd1* retinal sections treated with D- and L-cis-diltiazem (50 µM). DAPI (grey) was used as nuclear counterstain. While caspase-3 immunoreactivity was occasionally found in both outer and inner nuclear layer (ONL, INL), the percentage of caspase-3 positive cells was far lower than the numbers of dying cells (*cf*. Fig. 6). Scale bar = 50 µm.

21

Supplementary Figure S7

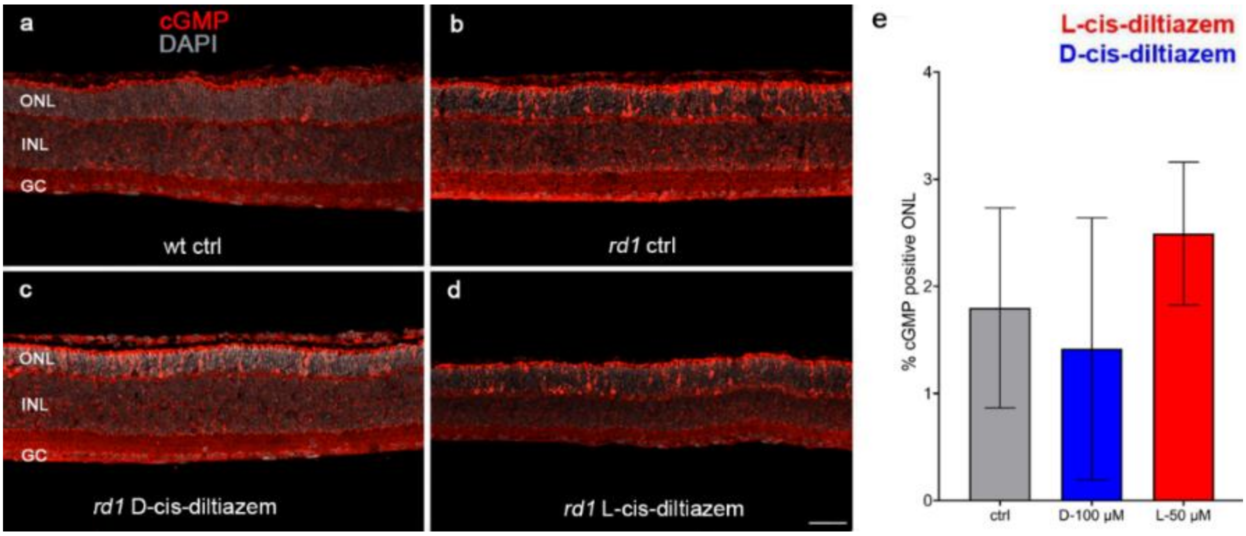

Figure S7: Accumulation of cGMP with/wo diltiazem treatment. Immunostaining for cGMP (red) was performed on wt and *rd1* retinal sections treated with D- and L-cis-diltiazem (50 µM). DAPI (grey) was used as nuclear counterstain. cGMP immunoreactivity was detected in photoreceptor segments and cell bodies in the outer nuclear layer (ONL). No significant change in the percentage of cGMP positive cells was observed neither with D-cis- nor with L-cis-diltiazem. Scale bar = 50 µm.

Supplementary Table S1

mV	cone CNGC														
	cGMP (µM)			+ 25µM D-cis-diltiazem			+100 µM D-cis-diltiazem			+25µM L-cis-diltiazem			+100 µM L-cis-diltiazem		
	EC_{50}	***H***	**n**	***EC_{50}***	***H***	**n**	***EC_{50}***	***H***	**n**	***EC_{50}***	***H***	**n**	***EC_{50}***	***H***	**n**
**-35**	26.0 ±2.9	1.81 ±0.1	7	31.7 ±1.6	1.71 ±0.1	6	28.8 ±1.4	1.54 ±0.1	7	31.1 ±1.1	1.36 ±0.09	5	42.6 ±4.0	1.28 ±0.1	6
**-100**	20.7 ±2.1	2.12 ±0.1	9	28.0 ±0.6	1.93 ±0.08	8	32.5 ±3.0	1.56 ±0.1	5	28.0 ±1.2	1.65 ±0.07	10	47.8 ±6.6	1.13 ±0.09	5
**+100**	13.5 ±3.2	1.70 ±0.1	5	27.5 ±1.7	1.43 ±0.1	7	49.9 ±6.0	1.20 ±0.05	5	40.6 ±4.9	1.13 ±0.1	9	56.1 ±9.9	0.95 ±0.1	5
	**rod CNGC**														
**-35**	70.1 ±5.3	1.74 ±0.1	7	85.7 ±8.8	1.71 ±0.1	7	95.4 ±9.2	1.68 ±0.1	6	79.2 ±8.1	1.49 ±0.2	5	103.2 ±12.4	1.28 ±0.1	6
**-100**	61.5 ±5.3	1.98 ±0.1	10	86.3 ±6.4	1.93 ±0.08	5	77.2 ±5.5	1.84 ±0.08	7	84.3 ±8.8	1.27 ±0.1	5	92.4 ±15.2	1.20 ±0.1	6
**+100**	46.5 ±6.5	2.02± 0.07	10	109.5 ±10.0	1.43 ±0.1	6	102.8 ±7.9	1.19 ±0.2	6	134.5 ±14.8	1.18 ±0.06	5	129.3 ±13.3	1.15 ±0.1	6

Table S1: Effect of D- and L-cis-diltiazem on the apparent affinity of rod and cone CNGCs. The EC_{50}-values and Hill coefficients (*H,* ±SEM) were obtained from the fit of the respective concentrations-activation relationships (n = number of experiments). Two-tailed unpaired Student *t*-test was used to compare the EC_{50}- and *H*-values in the presence of diltiazem with the ones obtained in its absence.

Supplementary Table S2

mV	Diltiazem Block (%) of cone CNGC at 3 mM cGMP					
	**+ 25 µM D-cis-diltiazem**	**+ 100 µM D-cis-diltiazem**	***p*-value**	**+ 25 µM L-cis-diltiazem**	**+ 100 µM L-cis-diltiazem**	***p*-value**
**-35**	8.37 ± 0.97	11.2 ± 1.1	0.0378	25.0 ± 1.6	43.2 ± 2.8	0.0002
**-100**	1.20 ± 0.3	8.02 ± 1.7	0.0001	4.9 ± 0.8	25.3 ± 3.4	<0.0001
**+100**	13.5 ± 1.6	34.0 ± 1.1	< 0.0001	39.2 ± 2.2	67.2 ± 1.4	<0.0001
	**Diltiazem Block (%) of rod CNGC at 3 mM cGMP**					
**-35**	31.5 ± 2.3	35.6 ± 0.9	ns	89.2 ± 0.37	91.5 ± 0.8	0.0270
**-100**	3.26 ± 1.7	11.9 ± 0.9	0.0006	52.4 ± 2.7	66.2 ± 2.0	0.0025
**+100**	53.5 ± 1.6	83.1 ± 1.2	<0.0001	90.0 ± 0.65	93.5 ± 1.4	0.0004
	**Diltiazem Block (%) of rod CNGC at 100 µM cGMP**					
**-35**	40.4 ± 3.4	46.1 ± 5.3	ns	88.4 ± 1.1	93.5 ± 1.1	0.0021

Table S2: Effect of D- and L-cis-diltiazem on the current amplitude of rod and cone CNGCs. The amount of block was determined by comparing the CNGC currents in the presence and in the absence of either D- or L-cis-diltiazem (±SEM, n=5-10) and was calculated using Eq. 2. The comparison between 25 and 100 µm of D- and L-cis-diltiazem, respectively, was performed using the two-tailed unpaired Student's *t*-test.

Supplementary Table S3

mV	cone CNGC:			*p*-value				
	cGMP + 25µM D-cis-diltiazem		cGMP + 100 µM D-cis-diltiazem		cGMP + 25µM L-cis-diltiazem		cGMP + 100 µM L-cis-diltiazem	
	EC_{50}	H	EC_{50}	H	EC_{50}	H	EC_{50}	H
**-35**	0.04242	ns	ns	ns	ns	0.00677	0.00277	0.00295
**-100**	0.00042	ns	0.000527	0.00934	0.000291	0.00121	0.000153	<0.0001
**+100**	0.000151	ns	0.00103	0.01494	0.00222	ns	0.01297	0.00636
	**rod CNGC:**			***p*-value**				
**-35**	ns	ns	0.02422	ns	ns	ns	0.02159	0.02111
**-100**	0.00534	ns	0.04734	ns	0.01729	0.00055	ns	0.00012
**+100**	<0.0001	<0.0001	<0.0001	<0.0001	<0.0001	<0.0001	<0.0001	<0.0001

Table S3: Statistical analysis of the effect of diltiazem on CNGC EC_{50}- and *H*-values at different voltages. The respective parameters and number of experiments are listed in Table S1. The EC_{50} and *H*-values in the presence of cGMP only were compared with the respective values in presence of cGMP and diltiazem.

Supplementary Table S4

	cone CNGC					
	τ_{act} (ms)	*p*-value	τ_{deact} (ms)	*p*-value	τ_{block} (ms)	*p*-value
**cGMP (µM)**	7.6 ± 2.1	-	52.1 ± 18.2	-		-
**+ 100 µM D-cis-diltiazem**	7.5 ± 2.6	ns	103.6 ± 39.1	0.0009	154.4 ± 53.4	ns
**+ 100 µM L-cis-diltiazem**	7.9 ± 2.8	ns	94.7 ± 36.8	0.0032	120.4 ± 38.5	
	**rod CNGC**					
**cGMP (µM)**	7.6 ± 2.1	-	52.1 ± 18.2	-		-
**+ 100 µM D-cis-diltiazem**	9.8 ± 2.6	ns	81.2 ± 30.5	0.0315	139.7 ± 60.2	ns
**+ 100 µM L-cis-diltiazem**	12.2 ± 4.1	ns	123.4 ± 46.1	0.0010	150.0 ± 56.7	

Table S4: Effect of D- and L-cis-diltiazem on the gating kinetics of cone and rod CNGCs. The effect of diltiazem on activation- and deactivation- time constants (τ_{act}, τ_{deact} and τ_{block}) in the presence of 3 mM cGMP (ms, ±SEM, n=5-9). Two-tailed unpaired Student *t*-test was used for the comparison between time constants obtained in the presence and in the absence of diltiazem.

Supplementary Table S5

Component	*p*-value	Effect-Size	ES-Lower-CI	ES-Upper-CI
**Model (all components)**		0.368	0.319	0.422
**AUC (control)**	<0.0001	0.195	0.147	0.247
**Treatment (drug)**	0.00123	0.05	0.023	0.085
**Treatment (concentration)**	0.3416	0.039	0.016	0.072
**AUC (control) x treatment (drug)**	0.119	0.003	0	0.017
**AUC (control) x treatment (conc.)**	0.171	0.002	0	0.015
**AUC (control) x treatment (drug) x treatment (conc.)**	<0.0001	0.021	0.005	0.047

Table S5: Effect of D- and L-cis-diltiazem on light-evoked Ca^{2+} signals in wt cone photoreceptors. The linear modelling identified the variables that significantly predict the data. The area-under-the-curve (AUC) in the control condition was significant and had the largest effect size, with semi-partial R-squared (SPRS) equal to 0.195 ($p < 0.0001$). The drug treatment and the drug concentration were both significant. There was also a statistically significant interaction between the AUC in the control condition, the drug treatment, and the drug concentration. Since their confidence intervals overlap, we cannot state which of these model components had the greatest effect size. There was neither a significant interaction between the AUC in the control condition and the drug treatment, nor between the AUC in the control condition and the drug concentration. (*cf*. Fig. 4).

Supplementary Table S6

	**OS length** n = 109, $R^2_{adj.}$ = 0.96		**ONL thickness** n = 109, $R^2_{adj.}$ = 0.93	
**Fixed effect**	***F*-statistic**	***p*-value**	***F*-statistic**	***p*-value**
genotype	*F*(1, 25.25 = 0.0078)	0.9304	*F*(1, 25.99) = 2.6450	0.1159
Time-point	*F*(5, 24.7) = 4.4213	0.0052	*F*(5, 24.77) = 15.1946	< 0.0001
Retinal position	*F*(2, 48.36) = 0.2982	0.7435	*F*(2, 49.39) = 2.9380	0.0623
genotype x time-point	*F*(5, 24.7) = 13.2699	< 0.0001	*F*(5, 24.77) = 12.0885	< 0.0001
genotype x retinal position	*F*(2, 48.36) = 0.3245	0.7245	*F*(2, 49.39) = 0.9156	0.4070
timepoint x retinal position	*F*(10, 47.94) = 3.8401	0.0007	*F*(10, 48.41) = 2.0258	0.0508
genotype x time-point x retinal position	*F*(10, 47.94) = 4.2248	0.0003	*F*(10, 48.41) = 1.3344	0.2397

Table S6: Analysis of the variability of OS length and ONL thickness in *rd1* and wt. Results of the linear mixed-effects models with the dependent variables OS length and ONL thickness. The models' residuals followed a normal distribution, while the Brown-Forsythe test indicated a violation of the assumption of homoscedasticity for both models. However, linear mixed-effects models estimates have been shown to be robust against such violations (81).

Supplementary Table S7

Dependent variable	Genotype	Fixed effect	Normality of residuals	Homo-scedasticity	*F*-statistic	*p*-value
TUNEL	wt (35) $R^2_{adj.}$ = .80 n = 336	Concentration[1]	Yes	No	*F*(3, 17.92) = 20.7656	<0.0001
		Treatment			*F*(1, 303.74) = 0.171	0.6795
		Concentration x Treatment			*F*(3, 22.8) = 29.6038	<0.0001
	rd1 (36) $R^2_{adj.}$ = .88 n = 331	Concentration[1]	Yes	No	*F*(3, 24.82) = 37.8570	< 0.0001
		Treatment			*F*(1, 306.63) = 0.0787	0.7792
		Concentration x Treatment			*F*(3, 27.75) = 31.0649	<0.0001
	rd10 (10) $R^2_{adj.}$ = .84 n = 112	Concentration[4]	Yes	No	*F*(1, 8.11) = 25.9134	<0.0009
		Treatment			*F*(1, 100.96) = 0.0026	0.9598
		Concentration x Treatment			*F*(1, 10.43) = 23.5461	<0.0006
Calpain activity	wt (21) & *rd1* (30) $R^2_{adj.}$ = .74 n = 51	Genotype	Yes	No	*F*(1, 45) = 29.1249	<0.0001
		Treatment			*F*(2, 45) = 56.1128	<0.0001
		Genotype x Treatment			*F*(2, 45) = 2.5937	0.0859
Calpain- 2	wt (9) & *rd1* (9) $R^2_{adj.}$ = .83 n = 117	Genotype	Yes	No	*F*(1, 12.14) = 9.0927	0.0106
		Treatment			*F*(2, 12.14) = 20.2775	0.0001
		Genotype x Treatment			*F*(2, 12.14) = 2.2535	0.1471
Caspase-3	*rd1* (11) $R^2_{adj.}$ = .04 n = 58	Treatment	Yes	Yes	*F*(2, 7.15) = 0.3799	0.6970
ONL localisation TUNEL	*rd1* (9) $R^2_{adj.}$ = .72 n = 53	Treatment	Yes	No	*F*(2, 10.11) = 49.4033	<0.0001
Treatment: (D-cis-diltiazem, L-cis-diltiazem), [1] (0, 25, 50, 100 µM), [2] (0, 25 µM), [3] (0, 50 µM), [4] (0, 100 µM)						

Table S7: Analysis of cell death markers using linear mixed-effects models. Shown are the effects that explain the variability of the dependent variables TUNEL, calpain activity, calpain-2 positive cells, as well as localization of TUNEL positive cells within the ONL. All models included the animal as a random effect to account for repeated measures. Numbers in brackets indicate the total number of animals used per genotype, n represents the number of observations used in the model. Normality of residuals was assessed visually; heterogeneity of residual variances (homoscedasticity) was tested with the Brown-Forsythe test. Linear mixed-effects models have been shown to be robust against violations of model assumptions (81).

Supplementary Table S8

	Contrast LS means [95% confidence interval] (%)		LS means diff. ± SE (%)	F-statistic	*p*-value
ONL localisation TUNEL	*rd1* ctrl 59.98 [54.04, 65.92]	*rd1* D-25 µM 57.44 [52.05, 62.83]	2.54 ± 3.79	F(1, 16.33) = 0.4511	0.5112
		rd1 L-25 µM 85.03 [79.70, 90.36]	25.05 ± 3.40	F(1, 10.42) = 54.2025	< 0.0001
Calpain activity	ctrl 2.41 [1.83, 3.00]	L-50 µM 6.42 [5.67, 7.17]	4.01 ± 0.47	*F*(1, 45) = 71.9711	<0.0001
Calpain-2	*rd1* ctrl 1.82 [1.20, 2.44]	*rd1* D-50 µM 0.68 [0.07, 1.30]	1.13 ± 0.40	F(1, 12.52) = 7.9008	0.0152
		rd1 L-50 µM 2.73 [2.11, 3.35]	0.91 ± 0.40	F(1, 12.69) = 5.0979	0.0423
	wt ctrl 0.52 [0.09, 1.13]	wt L-50 µM 2.04 [1.43, 2.66]	1.52 ± 0.39	F(1, 11.87) = 14.7372	0.0024
TUNEL	*rd1* ctrl 98.10 [50.88, 145.32]	*rd1* D-100 µM 180.85 [111.06, 250.63]	82.75 ± 41.14	F(1, 28.11) = 4.0454	0.0540
	rd1 ctrl 101.96 [54.54, 149.38]	*rd1* L-100 µM 661.96 [593.66, 730.26]	560.00 ± 40.99	F(1, 26.68) = 191.1994	<0.0001
	rd10 ctrl 90.68 [201.44, 382.81]	*rd10* D-100 µM 401.33 [111.58, 691.07]	310.60 ± 178.75	F(1, 8.10) = 3.0200	0.1200
	rd10 ctrl 93.60 [198.61, 385.80]	*rd10* L-100 µM 1403.14 [1060.80, 1745.48]	1310.00 ± 199.71	F(1, 9.25) = 42.9966	<0.0001
	wt ctrl 105.35 [58.38, 152.32]	wt D-100 µM 112.44 [43.49, 181.39]	7.10 ± 39.87	F(1, 19.17) = 0.0316	0.8607
	wt ctrl 98.28 [51.36, 145.21]	wt L-50 µM 458.14 [406.36, 509.93]	359.90 ± 33.31	F(1, 18.59) = 116.6931	<0.0001
		wt L-100 µM 420.42 [366.06, 474.78]	322.10 ± 34.59	F(1, 21.63) = 86.7207	<0.0001

Table S8: Post-hoc analysis of the linear mixed-effects models. Results of contrast tests comparing the least-square means, which resulted from the linear mixed-effects models shown in Table S7.

81. Schielzeth H, Dingemanse NJ, Nakagawa S, Westneat DF, Allegue H, Teplitsky C, et al. Robustness of linear mixed-effects models to violations of distributional assumptions. Methods Ecol Evol. 2020;11:1141–52.
