# Supplementary material for: Redefining the role of Ca2+-permeable channels in photoreceptor degeneration using diltiazem
Source: Cell Death Dis. 2022 Jan 10;13(1):47. doi: 10.1038/s41419-021-04482-1 (PMC8748460; doi:10.1038/s41419-021-04482-1)
Supplement: Supplementary file 5 — Related File [file 41419_2021_4482_MOESM5_ESM.pdf]

Gmail

Soumyaparna Das <soumyaparnadas@gmail.com>

Request agreement from all co-authors - CDDIS-21-1546R - manuscript Redefining the role of Ca2+-permeable channels in photoreceptor degeneration using diltiazem

16 messages

Soumyaparna Das <soumyaparnadas@gmail.com> 13 October 2021 at 13:41

To: Valerie.Popp@med.uni-jena.de, Michael Power <michaeljtpower@gmail.com>, kathrin.groeneveld@med.uni-jena.de, christian.melle@med.uni-jena.de, Luke Rogerson <luke.e.rogerson@gmail.com>, Marlly Natalia Achury <mnachurym@gmail.com>, fs@biolog.de, torsten.strasser@uni-tuebingen.de, Thomas Euler <thomas.euler@cin.uni-tuebingen.de>, Francois Paquet-Durand <francois.paquet-durand@klinikum.uni-tuebingen.de>, "Nache, Vasilica" <Vasilica.Nache@med.uni-jena.de>, jieyan19910809@hotmail.com

Dear all,

Thank you for your contribution to our joint manuscript CDDIS-21-1546R.
There is a request from the journal Cell Death & Disease for collecting a 'Request Agreement' from all co-authors of this manuscript CDDIS-21-1546R.

Please find in attachment, the final version of all the manuscript files (including all changes; additions and deletions) and please make sure to reply to this email as a confirmation if you agree to these changes.

All replies thus received from all the co-authors will be combined in one document and will be submitted to the journal.

Looking forward to your response.

With best regards,
Soumya

4 attachments

Das et al_CDDis_revised manuscript_citation edited_final.docx
250K

Supplementary Legends - Figures and Tables.docx
29K

Das et al-CDDis_Supplementary Figures and Tables_merged final.docx
1629K

Das et al-CDDis_Main text Figures_merged final.docx
1669K

Torsten Straßer <torsten.strasser@uni-tuebingen.de> 13 October 2021 at 13:44
To: Soumyaparna Das <soumyaparnadas@gmail.com>

Hi Soumya,

I agree with the manuscript and the submission.

All the best
Torsten
[Quoted text hidden]

Groeneveld, Kathrin <Kathrin.Groeneveld@med.uni-jena.de> 13 October 2021 at 13:44
To: Soumyaparna Das <soumyaparnadas@gmail.com>

Dear Soumya,

I agree with the changes in the attached manuscript.

Thank you for taking care of this request.

Bests

Kathrin Groeneveld

Dr. rer. nat. Kathrin Groeneveld

Innovationszentrum ThIMEDOP

Universitätsklinikum Jena

Nonnenplan 4

07743 Jena

Kathrin.Groeneveld@med.uni-jena.de

+49-3641-9 39 6604

[Quoted text hidden]

--
Universitätsklinikum Jena - Kastanienstraße1 - D-07747 Jena
Die gesetzlichen Pflichtangaben finden Sie unter https://www.uniklinikum-jena.de/UKJ_Pflichtangaben.html

---

Nache, Vasilica <VASILICA.NACHE@med.uni-jena.de> 13 October 2021 at 13:48
To: Soumyaparna Das <soumyaparnadas@gmail.com>

Hi Soumya,

this does not include the Editorial changes, isn't it?

If it is the same Version I read last time, than I agree with it.

Let me know when you have the Editorial proofs.

Bests

Vali

Dr. V. Nache
Institute of Physiology II
University Hospital Jena

Friedrich-Schiller University Jena
Kollegiengasse 9, 07743 Jena
Germany
Tel. +49-(0)3641-9397668
Fax +49-(0)3641-9397652
E-Mail : Vasilica.Nache@med.uni-jena.de

Von: Soumyaparna Das <soumyaparnadas@gmail.com>
Gesendet: Mittwoch, 13. Oktober 2021 10:11:22
An: Popp, Valerie; Michael Power; Groeneveld, Kathrin; Melle, Christian; Luke Rogerson; Marlly Natalia Achury; fs@biolog.de; torsten.strasser@uni-tuebingen.de; Thomas Euler; Francois Paquet-Durand; Nache, Vasilica; jieyan19910809@hotmail.com
Betreff: [ext] Request agreement from all co-authors - CDDIS-21-1546R - manuscript Redefining the role of Ca2+-permeable channels in photoreceptor degeneration using diltiazem

[Quoted text hidden]
[Quoted text hidden]

Soumyaparna Das <soumyaparnadas@gmail.com> 13 October 2021 at 13:58
To: "Nache, Vasilica" <VASILICA.NACHE@med.uni-jena.de>

Hi Vali,

You are correct, it does not include the editorial changes. It is the same final version from last time. They just want a written approval from all the co-authors. Could you please reply to that email again for them? Sure! I will update you with the Editorial proofs as soon as I receive them.

With best regards,
Soumya
[Quoted text hidden]

Nache, Vasilica <VASILICA.NACHE@med.uni-jena.de> 13 October 2021 at 14:04
To: Soumyaparna Das <soumyaparnadas@gmail.com>

What do you mean by:

"Could you please reply to that email again for them?"

Which email you mean? I did not receive any...

Bests

Vali

Dr. V. Nache
Institute of Physiology II
University Hospital Jena
Friedrich-Schiller University Jena
Kollegiengasse 9, 07743 Jena
Germany
Tel. +49-(0)3641-9397668
Fax +49-(0)3641-9397652
E-Mail : Vasilica.Nache@med.uni-jena.de

Von: Soumyaparna Das <soumyaparnadas@gmail.com>
Gesendet: Mittwoch, 13. Oktober 2021 10:28:38
An: Nache, Vasilica
Betreff: [ext] Re: [ext] Request agreement from all co-authors - CDDIS-21-1546R - manuscript Redefining the role of Ca2+-permeable channels in photoreceptor degeneration using diltiazem

[Quoted text hidden]

Soumyaparna Das <soumyaparnadas@gmail.com> 13 October 2021 at 14:38
To: "Nache, Vasilica" <VASILICA.NACHE@med.uni-jena.de>

Hi Vali,

I am sorry for the misunderstanding. By 'that email' I meant the email that I sent around (below). If you could please reply to that, I could make a document out of all the email responses from all the co-authors and send it to the journal as a 'Request Agreement. I am hoping that they will send us the Editorial proofs after that.

Dear all,

Thank you for your contribution to our joint manuscript CDDIS-21-1546R.
There is a request from the journal Cell Death & Disease for collecting a 'Request Agreement' from all co-authors of this manuscript CDDIS-21-1546R.

Please find in attachment, the final version of all the manuscript files (including all changes; additions and deletions) and please make sure to reply to this email as a confirmation if you agree to these changes.

All replies thus received from all the co-authors will be combined in one document and will be submitted to the journal.

Looking forward to your response.

With best regards,
Soumya

———

Thank you.

With best regards,
Soumya
[Quoted text hidden]

Nache, Vasilica <VASILICA.NACHE@med.uni-jena.de> 13 October 2021 at 14:45
To: Soumyaparna Das <soumyaparnadas@gmail.com>

Dear all,

I agree with the final version of the manuscript (CDDIS-21-1546R), including all changes, additions, and deletions resulting from the reviewer's comments.

All the best

Vasilica Nache

Dr. V. Nache
Institute of Physiology II

University Hospital Jena
Friedrich-Schiller University Jena
Kollegiengasse 9, 07743 Jena
Germany
Tel. +49-(0)3641-9397668
Fax +49-(0)3641-9397652
E-Mail : Vasilica.Nache@med.uni-jena.de

Von: Soumyaparna Das <soumyaparnadas@gmail.com>
Gesendet: Mittwoch, 13. Oktober 2021 10:11:22
An: Popp, Valerie; Michael Power; Groeneveld, Kathrin; Melle, Christian; Luke Rogerson; Marlly Natalia Achury; fs@biolog.de; torsten.strasser@uni-tuebingen.de; Thomas Euler; Francois Paquet-Durand; Nache, Vasilica; jieyan19910809@hotmail.com
Betreff: [ext] Request agreement from all co-authors - CDDIS-21-1546R - manuscript Redefining the role of Ca2+-permeable channels in photoreceptor degeneration using diltiazem

[Quoted text hidden]
[Quoted text hidden]

Francois Paquet-Durand <francois.paquet-durand@klinikum.uni-tuebingen.de> 13 October 2021 at 15:27
To: Valerie.Popp@med.uni-jena.de, Michael Power <michaeljtpower@gmail.com>, kathrin.groeneveld@med.uni-jena.de, christian.melle@med.uni-jena.de, Luke Rogerson <luke.e.rogerson@gmail.com>, Marlly Natalia Achury <mnachurym@gmail.com>, fs@biolog.de, torsten.strasser@uni-tuebingen.de, Thomas Euler <thomas.euler@cin.uni-tuebingen.de>, "Nache, Vasilica" <Vasilica.Nache@med.uni-jena.de>, jieyan19910809@hotmail.com, Soumyaparna Das <soumyaparnadas@gmail.com>

Dear Soumya,

Thank you for sending these files around and for your all your efforts to finally make publication possible.

I herewith confirm that I do agree to the publication in CDDis of our manuscript entitled:
Redefining the role of Ca^{2+}-permeable channels in photoreceptor degeneration using diltiazem.

with kind regards,
François Paquet-Durand
--
Prof. Dr. rer. nat. F. Paquet-Durand
Cell Death Mechanism Group
Institute for Ophthalmic Research
University of Tuebingen
Elfriede Aulhorn Straße 5-7, 72076 Tuebingen, Germany

e-mail: francois.paquet-durand@uni-tuebingen.de
Skype: francois.paquet-durand
Phone: +49 7071 29 87430
Fax: +49 7071 29 5777

[Quoted text hidden]
Erstellt mit Operas E-Mail-Modul: http://www.opera.com/mail/

Thomas Euler <thomas.euler@cin.uni-tuebingen.de> 13 October 2021 at 15:51
To: Soumyaparna Das <soumyaparnadas@gmail.com>, Valerie.Popp@med.uni-jena.de, Michael Power <michaeljtpower@gmail.com>, kathrin.groeneveld@med.uni-jena.de, christian.melle@med.uni-jena.de, Luke Rogerson <luke.e.rogerson@gmail.com>, Marlly Natalia Achury <mnachurym@gmail.com>, fs@biolog.de, torsten.strasser@uni-tuebingen.de, Francois Paquet-Durand <francois.paquet-durand@klinikum.uni-tuebingen.de>, "Nache, Vasilica" <Vasilica.Nache@med.uni-jena.de>, jieyan19910809@hotmail.com

Dear Soumya,

I agree with the revision of the manuscript.

Great job!

Thanks and best
Thomas

Am 13.10.2021 um 10:11 schrieb Soumyaparna Das:
[Quoted text hidden]

严 劼 <JIEYAN19910809@hotmail.com> 13 October 2021 at 16:23
To: Soumyaparna Das <soumyaparnadas@gmail.com>

Dear Soumya

Thank you for your efforts. I agree to these changes.

Best
Jie Yan

发件人: Soumyaparna Das <soumyaparnadas@gmail.com>
发送时间: 2021年10月13日 16:11
收件人: Valerie.Popp@med.uni-jena.de <Valerie.Popp@med.uni-jena.de>; Michael Power <michaeljtpower@gmail.com>; kathrin.groeneveld@med.uni-jena.de <kathrin.groeneveld@med.uni-jena.de>; christian.melle@med.uni-jena.de <christian.melle@med.uni-jena.de>; Luke Rogerson <luke.e.rogerson@gmail.com>; Marlly Natalia Achury <mnachurym@gmail.com>; fs@biolog.de <fs@biolog.de>; torsten.strasser@uni-tuebingen.de <torsten.strasser@uni-tuebingen.de>; Thomas Euler <thomas.euler@cin.uni-tuebingen.de>; Francois Paquet-Durand <francois.paquet-durand@klinikum.uni-tuebingen.de>; Nache, Vasilica <Vasilica.Nache@med.uni-jena.de>; jieyan19910809@hotmail.com <jieyan19910809@hotmail.com>
主题: Request agreement from all co-authors - CDDIS-21-1546R - manuscript Redefining the role of Ca2+-permeable channels in photoreceptor

degeneration using diltiazem

[Quoted text hidden]

Frank Schwede, BIOLOG LSI <fs@biolog.de> 13 October 2021 at 16:29
To: Soumyaparna Das <soumyaparnadas@gmail.com>
Cc: Valerie.Popp@med.uni-jena.de, Michael Power <michaeljtpower@gmail.com>, kathrin.groeneveld@med.uni-jena.de, christian.melle@med.uni-jena.de, Luke Rogerson <luke.e.rogerson@gmail.com>, Marlly Natalia Achury <mnachurym@gmail.com>, torsten.strasser@uni-tuebingen.de, Thomas Euler <thomas.euler@cin.uni-tuebingen.de>, Francois Paquet-Durand <francois.paquet-durand@klinikum.uni-tuebingen.de>, "Nache, Vasilica" <Vasilica.Nache@med.uni-jena.de>, jieyan19910809@hotmail.com

Dear Soumya,

Thank you for the files around and and all your efforts!

I herewith confirm that I do agree to the publication in CDDis of our manuscript entitled:
Redefining the role of Ca^{2+}-permeable channels in photoreceptor degeneration using diltiazem.

Best regards,
Frank

____
Frank Schwede, PhD
General Manager Operations / Head of R & D
Biolog Life Science Institute GmbH & Co. KG
Flughafendamm 9a
28199 Bremen
Germany
Phone:+49 (0)421 591355
Fax: +49 (0)421 5979713
E-mail: fs@biolog.de
Web: https://www.biolog.de/

Terms and Conditions of Sale and Synthesis:
https://www.biolog.de/business_terms
Allg. Geschäftsbedingungen für Verkauf und Synthesen:
https://www.biolog.de/agb
Privacy Policy: https://www.biolog.de/privacypolicy
Datenschutzerklärung: https://www.biolog.de/datenschutzerklarung

Amtsgericht Bremen, HRA 28468 HB
USt-IdNr./VAT number: DE324702823
Persönlich haftende Gesellschafterin: Biolog Verwaltungs GmbH, Bremen
Amtsgericht Bremen, HRB 34454 HB
Geschäftsführer/General Managers: Andrea Intemann, Dr. Frank Schwede

Please note that any e-mail sent to fs@biolog.de may be read and handled by another Biolog Life Science Institute representative in case the intended recipient is absent. Please be assured that all information submitted will be treated as strictly confidential.
This message, and any attachment, is intended only for the use of the individual or entity to which it is addressed, and may contain information that is privileged, confidential, and/or exempt from disclosure by applicable law or court order. If the reader of this message is not the intended recipient, you are hereby notified that any use, dissemination, distribution, or copying of this communication is strictly prohibited. If you have received this communication in error, please notify us immediately by return e-mail. Please also immediately delete the message from your computer system. Thank you.
[Quoted text hidden]

Melle, Christian <CHRISTIAN.MELLE@med.uni-jena.de> 13 October 2021 at 17:26
To: Soumyaparna Das <soumyaparnadas@gmail.com>

Dear Soumya,

I agree with the revision of our manuscript (CDDIS-21-1546R) accepted by Cell Death & Diseases.

Best regards,

Christian

PD Dr. Christian Melle
Biomolecular Photonics Group
Universitätsklinikum Jena
Nonnenplan 4
D-07743 JENA
Germany
phone:+49 3641 9 397807
fax:+493641 9 396602
Email:CHRISTIAN.MELLE@med.uni-jena.de

Von: Soumyaparna Das <soumyaparnadas@gmail.com>
Gesendet: Mittwoch, 13. Oktober 2021 10:11:22
An: Popp, Valerie; Michael Power; Groeneveld, Kathrin; Melle, Christian; Luke Rogerson; Marlly Natalia Achury; fs@biolog.de; torsten.strasser@uni-tuebingen.de; Thomas Euler; Francois Paquet-Durand; Nache, Vasilica; jieyan19910809@hotmail.com

Betreff: [ext] Request agreement from all co-authors - CDDIS-21-1546R - manuscript Redefining the role of Ca2+-permeable channels in photoreceptor degeneration using diltiazem

[Quoted text hidden]
[Quoted text hidden]

Marlly Natalia Achury <mnachurym@gmail.com> 13 October 2021 at 18:34
To: Soumyaparna Das <soumyaparnadas@gmail.com>

Dear Soumya,

Thanks for coordinating the process and all your effort to publish this manuscript!
I fully agree with the content and the changes done to get the final version of it.

Best,
Marlly
[Quoted text hidden]

Michael Power <michaeljtpower@gmail.com> 15 October 2021 at 02:16
To: Soumyaparna Das <soumyaparnadas@gmail.com>

Hey Soumya,

I agree, publish away.

[Quoted text hidden]

Luke Rogerson <luke.e.rogerson@gmail.com> 20 October 2021 at 00:18
To: "Frank Schwede, BIOLOG LSI" <fs@biolog.de>
Cc: Soumyaparna Das <soumyaparnadas@gmail.com>, Valerie.Popp@med.uni-jena.de, Michael Power <michaeljtpower@gmail.com>, kathrin.groeneveld@med.uni-jena.de, christian.melle@med.uni-jena.de, Marlly Natalia Achury <mnachurym@gmail.com>, Torsten Straßer <torsten.strasser@uni-tuebingen.de>, Thomas Euler <thomas.euler@cin.uni-tuebingen.de>, Francois Paquet-Durand <francois.paquet-durand@klinikum.uni-tuebingen.de>, "Nache, Vasilica" <Vasilica.Nache@med.uni-jena.de>, jieyan19910809@hotmail.com

Dear Soumya

I confirm that I am happy with the changes to the manuscript and consent for this version to be published.

Kind regards,
Luke Edward Rogerson
[Quoted text hidden]

Gmail

Soumyaparna Das <soumyaparnadas@gmail.com>

Request agreement from all co-authors - CDDIS-21-1546R - manuscript Redefining the role of Ca2+-permeable channels in photoreceptor degeneration using diltiazem

1 message

Valerie Popp <Vali-Laura@gmx.de> 13 October 2021 at 17:53
To: soumyaparnadas@gmail.com

Dear Soumya,

I herewith confirm that I do agree to the publication in CDDis of our manuscript entitled:

Redefining the role of Ca^{2+}-permeable channels in photoreceptor degeneration using diltiazem."

Best regards

Valerie Popp
