# Supplementary material for: Redefining the role of Ca2+-permeable channels in photoreceptor degeneration using diltiazem
Source: Cell Death Dis. 2022 Jan 10;13(1):47. doi: 10.1038/s41419-021-04482-1 (PMC8748460; doi:10.1038/s41419-021-04482-1)
Supplement: Supplementary file 6 — Related File [file 41419_2021_4482_MOESM6_ESM.docx]

**Author Contributions**

Das *et al*.: Redefining the role of Ca^2+^-permeable channels in photoreceptor degeneration using diltiazem.

S. Das performed retinal explant cultures, TUNEL and immunostaining, microscopy, analysed data and helped write the manuscript; M. Power performed Ca^2+^-imaging experiments; V. Popp studied the effect of diltiazem on CNGC gating kinetics; K. Groeneveld performed colocalization experiments for heterotetrameric CNGCs; C. Melle performed molecular-biology work; J. Yan performed immunostaining; M. Achury performed immunostaining and analysed data; L. Rogerson analysed Ca^2+^-imaging data and performed statistical analysis; T. Strasser performed statistical analysis on immune- and bioassay data; F. Schwede synthesized fluorescent cGMP derivatives; V. Nache performed electrophysiological and optical measurements to study the effect of diltiazem on CNGC; V. Nache, T. Euler, and F. Paquet-Durand designed the experiments, interpreted the data, and prepared the manuscript. All authors edited the manuscript.
